# Event and Comment

**Published:** 1916-06

**Authors:** 


### DENTAL REGISTER

Vol. LXX JUNE, 1916 No. 6

### EVENT AND COMMENT

LEGALIZED DENTAL HYGIENISTS. The agitation for preventive measures in Oral Hygiene has resulted in a demand for a new service to the public, which the state of New York is the first to authorize on a legal basis. The new statute regulating the practice of dentistry in that state authorizes the education of persons who may practice to a limited extent oral prophylactic measures. Any legally incorporated dental infirmary may instruct students and grant diplomas certifying their qualifications, and these diplomas will entitle their holders to be registered and become legalized practitioners of certain phases of oral prophylaxis. It is the intention of the new Eastman Dispensary, at Rochester, to take up this work. This will insure the proper and adequate preparation of these practitioners, as we are assured that no one who is incompetent will be graduated from such an institution. This is a most promising solution of a question which has aroused much discussion in the profession, and has been looked upon with suspicion that it would be the cause of much illegal practice. Undoubtedly other institutions will take up this work and it will be fortunate for the cause if all such training may be of such a character as to inspire confidence with the profession and public. There can be little question as to the necessity for these practitioners. There is a vast need not only in our public schools, but children who are still too young to be in school, children in hospitals and institutions, and even hospital patients of every age can be cared for as by no other means at present available. The field is enormous and it will take a long time to educate enough hygienists to take care of this public service, to say nothing of the work demanded in connection with private practices. The realization of this idea in so satisfactory a manner is a matter of much importance to the profession as well as the public, and we are indebted to New York State for inaugurating so satisfactory a method of training the oral hygienist. Other states will undoubtedly enact similar laws or at least amend their laws regulating practice so as to permit such people to register and practice this form of oral hygiene. If as much care and thought is expended on the curriculum and on its administration through properly qualified institutions we shall have good reason to look forward to better progress in the field of preventive dentistry.

In connection with this subject it may be interesting to record that the Oral Hygienists of Connecticut have just held a convention in connection with the Connecticut State Dental Society and the program presented there would do credit to any dental society. The principal address on the peridental membrane was made by Dr. F. C. Noyes, of Chicago. There were three other papers and very good discussions besides clinics. It looks very much as though we are to have another useful department added to our profession. There was exhibited in this convention a degree of enthusiasm and devotion that promises a bright future for educated hygienists.

